# Congruent biogeographical disjunctions at a continent-wide scale: Quantifying and clarifying the role of biogeographic barriers in the Australian tropics

**DOI:** 10.1371/journal.pone.0174812

**Published:** 2017-04-04

**Authors:** Robert D. Edwards, Michael D. Crisp, Dianne H. Cook, Lyn G. Cook

**Affiliations:** 1 School of Biological Sciences, The University of Queensland, Brisbane, Queensland, Australia; 2 Research School of Biology, The Australian National University, Acton, Australian Capital Territory, Australia; 3 Department of Econometrics and Business Statistics, Monash University, Clayton, Victoria, Australia; Institute of Botany, CHINA

## Abstract

**Aim:**

To test whether novel and previously hypothesized biogeogaphic barriers in the Australian Tropics represent significant disjunction points or hard barriers, or both, to the distribution of plants.

**Location:**

Australian tropics: Australian Monsoon Tropics and Australian Wet Tropics.

**Methods:**

The presence or absence of 6,861 plant species was scored across 13 putative biogeographic barriers in the Australian Tropics, including two that have not previously been recognised. Randomizations of these data were used to test whether more species showed disjunctions (gaps in distribution) or likely barriers (range limits) at these points than expected by chance.

**Results:**

Two novel disjunctions in the Australian Tropics flora are identified in addition to eleven putative barriers previously recognized for animals. Of these, eleven disjunction points (all within the Australian Monsoon Tropics) were found to correspond to range-ending barriers to a significant number of species, while neither of the two disjunctions found within the Australian Wet Tropics limited a significant number of species’ ranges.

**Main conclusions:**

Biogeographic barriers present significant distributional limits to native plant species in the Australian Monsoon Tropics but not in the Australian Wet Tropics.

## Introduction

Climatic and geographic barriers can play a major role in the diversification of organisms across landscapes by limiting species’ distributions, dispersal and gene flow. Identifying such barriers has the potential to contribute to explanations of the geographic limits of species and, ultimately, the genesis of species when isolation is sustained for prolonged periods of time. Strong and persistent barriers are particularly useful for understanding patterns of biogeography and speciation because they are expected to influence the histories of multiple species in similar ways [1,2]. Physical features with sharply defined borders (e.g. oceans, rivers, mountain ranges) are obvious candidates, however identifying and defining putative barriers when no obvious geophysical features are apparent, or where different species recognize the same barrier-edge along something of a gradient (as may be expected when climatic or geochemical factors are in play), can be difficult. Potential barriers can be detected indirectly by finding congruent breaks in the distributions of multiple species, especially if such organisms have similar ecological tolerances and are not closely related [3].

**Fig 1:**
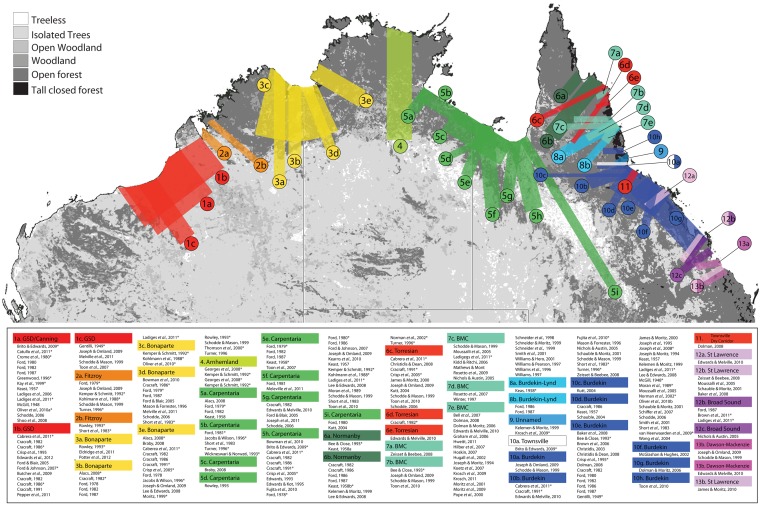
Diagrammatic summary of the variation in placement and size of biogeographic barriers previously illustrated or described in the Australian tropics. Coloured blocks represent gaps or points of disjunction between species and populations, and typically extend from the coast towards the center of the continent. References for each putative barrier are provided in the table (see S1 Document for full references), with asterisks denoting where barriers are unnamed, or described but not explicitly illustrated. The underlying vegetation structure map is modified from the Joint Remote Sensing Project (persistent URL: http://auscover.org.au/purl/icesat-vegetation-structure) and is used under a CC BY 4.0 License.

In Australia, up to 22 major zones of coincident turnover or common disjunction in species’ distributions have been identified as putative barriers to dispersal, primarily in vertebrates (e.g., [4,5,6]) and, while the timing and underlying causes have been robustly inferred for some (e.g., the uplift of the Nullarbor Plain dividing the temperate regions of southeast and southwest Australia [7], and the periodic connection and separation of Tasmania from the mainland [8,9]), most remain poorly defined and largely untested. Observing that many of these putative barriers coincide roughly with climatic and/or topographic features, authors have often invoked these as the processes causing—or having caused—divergence (e.g., [10,4,11,12]) despite little empirical evidence. Furthermore, putative barriers have had different names applied, or have been placed in differing locations (Fig 1), leading to confusion in comparisons across studies [13]. Defining a stable framework that uses consistent locations, names, and definitions for putative biogeographic barriers is essential for constructing and testing hypotheses about the causes of these phenomena, as well as their role in speciation and community assembly [14].

### Biogeography of the Australian tropics

**Fig 2:**
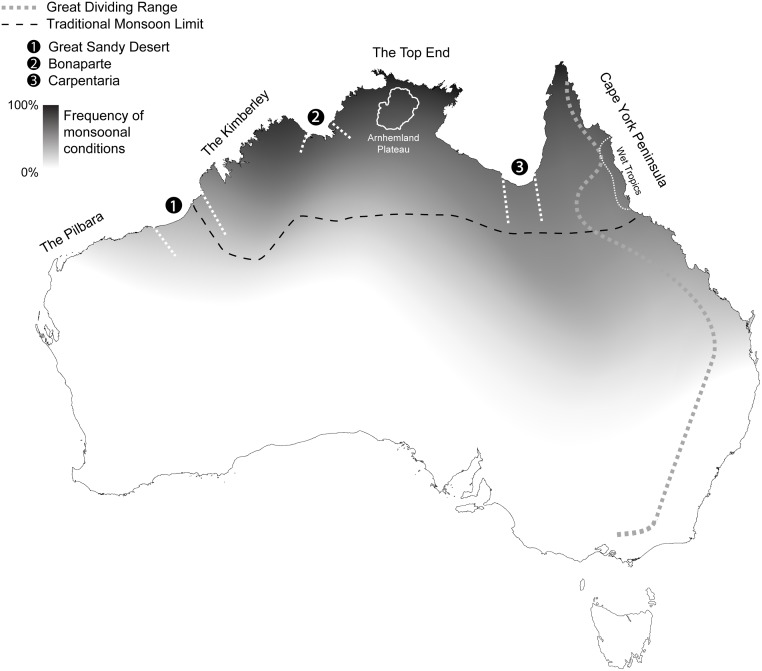
The Australian continent with shading indicating the frequency of monsoonal conditions (modified from [17]). Black dashed line indicates typically recognised southern limit of the monsoon zone. White dashed lines mark the three largest putative biogeographic barriers used as the a priori seed for this study, and thought to divide the monsoon tropics into four main regions (The Pilbara, The Kimberley, The Top End, and Cape York Peninsula). The Arnhemland Plateau, Wet Tropics and Great Dividing Range are also indicated. The map outline was modified from Geoscience Australia (http://www.ga.gov.au/metadata-gateway/metadata/record/61754/) and is used under a CC BY 4.0 license.

The Australian Tropics (also Australian Tropical Zone [15]) comprises two distinct biomes, the extensive Australian Monsoon Tropics (AMT) and a small mosaic of highly mesic habitats that makes up the Australian Wet Tropics (AWT) (Fig 2). Together, these cover about one third of the Australian continent and carry a proportional fraction of described native plant species (about 6,800 of approximately 20,000). While up to 11 putative biogeographic barriers have been identified in the distributions of animals across the Australian Monsoon Tropics and Australian Wet Tropics [4,13], only one is well characterized using disparate taxa: studies on the Black Mountain Corridor region indicate that some plant and animal species have been subjected to multiple bouts of vicariance correlated with aridification and habitat fragmentation at glacial maxima [16]. Few studies have attempted to provide a broader synthesis, with most modern molecular work invoking barriers ad- or post-hoc and only considering the distribution of one or few taxa. The role of putative barriers in shaping the distributions of plants across northern Australia has been generally neglected.

In this study, we compare the distributions of 6,861 plant species found in the Australian Tropics in order to determine whether plants across the Australian Tropics share common disjunctions, and to identify which of these disjunctions are congruent with those cited in previous studies. We address the question of whether the barriers could be real by testing whether the number of plant species disjunct across each putative biogeographic barrier is greater than expected by chance. We then address the question of whether the barriers are so severe that they represent range ends by testing whether plant species’ distributions end at a barrier more frequently than expected by chance. We also review and describe each putative barrier while attempting to resolve confusion around the naming and placement of putative barriers in previous studies.

## Methods

### Defining the Australian Monsoon tropics

Our dataset includes all Australian plant species that frequently encounter monsoonal conditions within the Australian continent (>85% of annual rainfall occurring between November and April, inclusive [12]) and those in the Australian Wet Tropics. Although not typically considered as part of the Australian Monsoon Tropics, we include the Pilbara (Western Australia) and coastal central Queensland because these areas experience monsoon-like rainfall patterns, even if rarely (Fig 2, modified from [17]), and because many plant species occurring in the traditionally recognised Australian Monsoon Tropics also occur in these regions.

The Australian Wet Tropics dataset includes the area along the east coast of Australia between Cooktown (-15° 28’ S) and Ingham (-18° 38’ S) and, although small fragments of wet tropical habitat are found further south, these are embedded in drier forest and are not considered part of the Australian Wet Tropics for this study.

### Data assembly

Checklists of plant species were obtained for the three Australian states/territories that fall within the study areas: the Northern Territory (Checklist of NT Vascular Plant Species [18]); Queensland [19,20]; and Western Australia [21]). Lists were cross-referenced to eliminate duplicates and synonyms.

Where applicable, taxon names were checked against the Australian Plant Name Index [22] and relevant taxonomic papers were consulted if further clarification was needed. 9,101 plant species were identified as occurring within these three administrations, of which 2,240 were excluded as having ranges that do not fall within the Australian Monsoon Tropics or Australian Wet Tropics as defined above, as having insufficient locality data, or as being introduced exotics. It should be noted that most of the remaining 6,861 species are recognised on the basis of morphological classifications alone and future molecular investigation might reveal cryptic species not considered here.

Geographic outliers (isolated collections falling well outside the main distribution of a species’ collection records) were checked against state checklists, the Flora of Australia [23] and Flora of Australia online where available [24] and in primary literature, and removed if unverified.

### Data scoring

There is considerable inconsistency in the number and location of putative barriers previously invoked across northern Australia (Fig 1). In an attempt to identify disjunctions, without confirmation bias, we used an agnostic iterative process whereby the distributions of an initial subset of 1,000 random species were scored for presence or absence of disjunctions across three major landscape features (Great Sandy Desert, Bonaparte Gulf, Carpentaria Basin). During this process the presence of further disjunctions was noted. The 1,000 taxa were re-scored to include these new disjunctions while refining their locations, and any further shared gaps were noted. Five iterations of gap discovery were conducted on this initial dataset leading to the identification of 13 disjunctions common across many taxa. Only subsequently were these disjunctions compared to putative barriers previously identified in the literature.

Distribution maps were generated for each of the 6,861 plant species using the Australian Virtual Herbarium [25]. The distribution of each species was scored with respect to the 13 putative biogeographic barriers identified in the initial training set (11 in the Australian Monsoon Tropics and two in the Australian Wet Tropics). Presence or absence was recorded for each species within the putative biogeographic barrier and also in each of the two flanks. Thus, for the region of each putative biogeographic barrier, we scored three binary variables. For example, a "disjunct" species, which is present on both sides but not within a putative barrier, was scored 101. A species present across the region and showing no apparent disjunction was coded 111; a species that is absent from the putative barrier and its flanks was coded 000; and so on. For those flanking regions where records were sparse (< 5 locality records per region) presence/absence was recorded as unknown. All such species with unknown occurrence data in the region of a given barrier were removed from the dataset prior to any statistical test relevant to that barrier (below). A spreadsheet of the data is available online via Mendeley Data (at: http://dx.doi.org/10.17632/8vgv4zzhhg.2).

**Fig 3:**
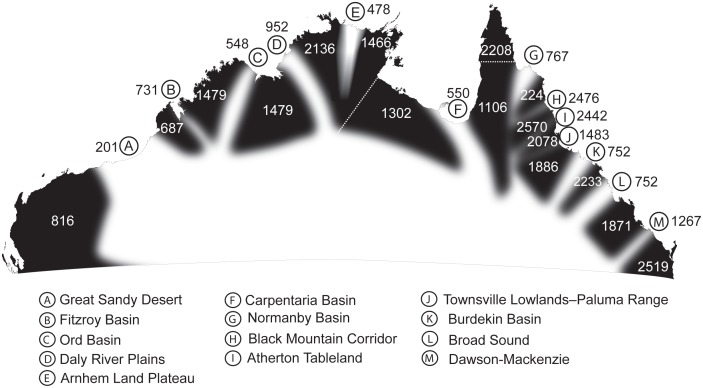
The thirteen shared disjunctions identified in this study. Circled letters denote zones of common disjunction. Numbers indicate species recorded in each putative region of disjunction and associated flanking regions. White dotted lines indicate the divisions in flanking regions applied between the Carpentaria Basin and the Arnhem Land Plateau, and between the Carpentaria Basin and the Normanby Basin.

Because of the disparity in climate and vegetation types between the Arnhem Land Plateau and Carpentaria Basin, and between the Carpentaria Basin and the Normanby Basin, these two flanking regions were more narrowly defined (dotted lines in Fig 3) to reduce potential bias in comparisons of flanking regions and putative barriers.

### Pattern and significance of species’ responses to putative barriers

The distributional data were used to test two hypotheses. Firstly, we tested the hypothesis that barriers are impermeable; that is, a species occurring in the region of a barrier (but not in the barrier) is likely to occur in one flank or the other, but not in both. Each barrier was assessed in turn and occurrences outside its flanks were ignored. Permutation testing was conducted with the following steps using R v3.3.2 in RStudio v0.99.902 (script provided in supplementary material via Mendeley data: http://dx.doi.org/10.17632/thcpyyy7br.1):

1. Subset data for each barrier to include only species present in either or both flanks of the barrier. Also remove species that have an unknown value in the barrier column.
2. Calculate the proportion of each of the patterns “100”, “001”, “101” for the actual data (patterns are in the order of the columns in the spreadsheet lodged online).
3. Permute column 2 and 3 independently, breaking any association between presence/absence of the barrier and the two flanks. Record the frequency of each pattern. Repeat 5000 times.
4. Compute the proportions for the patterns for each permuted data set, and record the 2.5^th^ and 97.5^th^ percentiles which provides a 95% confidence interval for the expected occurrence of the pattern, assuming that the proportion of times the patterns occur is purely random.
5. If the actual proportion is lower than the interval, then it is lower than expected, and conversely is higher than expected if it is larger than the interval. “001”, “100” represent impermeable barrier patterns, and “101” would suggest permeable.
6. Repeat the above but holding column 3 while permuting 1 and 2 to ensure robustness of results across alternative permutations.

We also conducted a series of the above tests for the Great Sandy Desert barrier using increasing numbers of permutations to assess stationarity of the results. No fluctuations were observed above 1000 replicates, and 5000 replicates used in the final analyses for surety (see http://dx.doi.org/10.17632/8vgv4zzhhg.2 for results).

**Fig 4:**
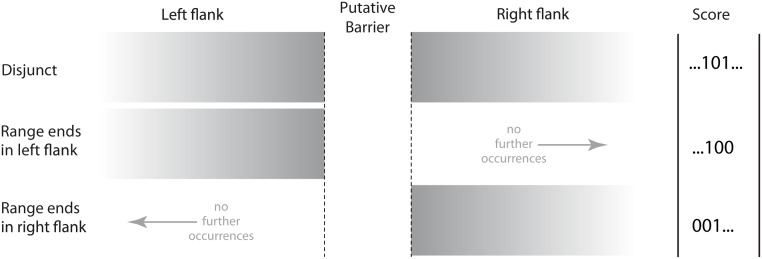
Disjunct and range-end patterns for species in or adjacent to putative barriers, and how they were scored. Shaded regions represent occurrence of one species.

Secondly, we asked whether species’ ranges end at barriers more frequently than expected by chance. In other words, do species’ ranges tend to stop at the flanks of barriers and extend no farther, either into the barrier itself or to the far side of the barrier? Under the first hypothesis, described above, the “barrier impermeable” category included some species that occurred only in one flank of the barrier in question but extended further away, beyond the flank on the far side of the barrier. Distributions of this kind, that did not end in the region of the barrier, were excluded from the second test. Using the same set of permuted data as for the first question, we counted the relative proportion of ranges that ended in a flank (…100) versus in a barrier (…110 or …010), i.e. in this example the range ends at the left flank (Fig 4). For testing whether the range ends at a right flank, we compared the converse patterns, i.e. (001…) versus (011… or 010…). We used the binomial test to evaluate whether these proportions are different from random, with the null expectation that 33% of distributions should end in a flank, i.e., with the pattern “001…” or “…100”. The tests were implemented using the SISA website as "SISA-Binomial” [26].

## Results

### Common disjunctions

Thirteen common disjunctions were identified across the Australian Tropics from the distributions of the 6,861 plant species (Fig 3). In 11 cases these can be broadly matched to gaps previously described in the distributions of animal species. From west to east these are: Great Sandy Desert (GSD); Fitzroy Basin, Ord Basin, Daly River Plains, Arnhem Land Plateau, Carpentaria Basin, Normanby Basin, Black Mountain Corridor, Atherton Tableland, Townsville Lowlands-Paluma Range, Burdekin Basin, Broad Sound, and Dawson-Mackenzie. Precise spatial definition of barrier boundaries is problematic due, in part, to the resolution of the spatial data—species have not been sampled exhaustively (or evenly)–and also to the likelihood that individual species boundaries are not fully congruent. This is likely to be particularly true where putative barriers are part of an environmental gradient, for example, the aridity gradient leading into the Great Sandy Desert. While noting that the exact delimitation of barriers is problematic, the two narrowest putative barriers identified in this study were both within the Australian Wet Tropics (Atherton Tableland ~30 km wide; Black Mountain Corridor ~50–60 km wide).

We provide a description of each putative barrier including estimated width, location, taxonomy, and notes on conflicting descriptions from previous studies in Table 1. In the many cases where several names have been used for what appear to be the same region, we use the name of the regional landform. A comprehensive map of previous demarcations of each barrier can be found in Fig 1 with an associated list of references. In the Australian Monsoon Tropics, all but one of the putative barriers are dry corridors compared with their flanking regions, sometimes also with differing soil type (Table 1). The exception is the Arnhem Plateau, newly recognized here as a region associated with plant disjunctions, and which is a dissected escarpment rather than a drier region. One region of disjunction in the Australian Wet Tropics is a drier corridor (BMC, Table 1) whereas the other, the newly recognized Atherton Tableland, is a region of high elevation.

**Table 1:** Description and notes on the 13 putative barriers identified in this study, as illustrated in Fig 3.

Putative barrier and description
***A. Great Sandy Desert (GSD, also Canning Gap*** [27]***)*** *Location in this study*: Approximately 390 km wide where intersecting the coast between the De Grey River (east of Port Hedland) and Roebuck Bay and Plains (immediately south of Broome), and extending inland to the SE. The northern edge extends linearly SE with no obvious bounding feature, while in the south expansion is limited by the Pilbara craton. *Notes*: Well recognized as a major barrier between the Pilbara and Kimberley regions, often implicated in the isolation of species [10,28] and populations [29]. Despite being commonly recognized, the bounds of this barrier are often poorly defined. Beginning with the original description [27], most references to this barrier use generalized or stylized diagrams that vary considerably in size, location, and orientation.
***B*. *Fitzroy Basin (also West Fitzroy Barrier***[4]***)*** *Location in this study*: A 110 km gap in the Kimberley, running NW-SE from the head of King Sound and following the Lennard Shelf between the Fitzroy River and the King Leopold Ranges. Roughly parallel to the GSD. *Notes*: A little-recognized offshoot of the Great Sandy Desert comprising flat exposed sediments and sand plains associated with the Fitzroy River [30], this separates elements of the South Kimberley Pindan region from the North Kimberley Division [31].
***C. Ord Basin (also Bonaparte Gap*** [32,10], ***East-West Kimberley Divide*** [33]***)*** *Location in this study*: An approximately 120 km wide belt between Cape Rulhieres and Cambridge Gulf, running slightly west of N-S towards (and effectively joining) the Fitzroy Basin and GSD. *Notes*: A number of biogeographic breaks have been identified in the region of the Bonaparte Gulf [10,13] leading to confusion surrounding their naming and placement across various studies and with many authors nominating only a single larger gap [12,34,35,36]. Eldridge *et al*., [13] provide a useful review of this confusion, concluding that there are five distinct biogeographic barriers: The Ord Arid Intrusion, Victoria River Drainage, Daly River Drainage, and Bonaparte Gap as well as suggesting that a new putative barrier—the “East West Kimberley Divide”—isolates genetic groups of rock wallabies [37]. How this new gap is distinct from an extension of Ford’s Bonaparte Gap (previously only recognized in mangrove taxa [32,33]) is unclear, and we suggest the two may be one and the same. Our data identified two disjunctions in plant distributions within this region. The first is the more westerly and is the E-W Kimberley Divide/Bonaparte Gap [32,33] (not to be confused with a broader definition indicated by the same author in 1987[13]). The second is the Daly River Plains—see below. Interestingly there is no obvious physical delimitation of this gap and while it is suggested that a basalt range running north-south may provide habitat disruption [37] this is not entirely congruent with the disjunctions observed here. A closer association may be found with a wide band of Pentecost and Warton sandstones, although how these differ from the surrounding King Leopold sandstone is unclear.
***D. The Daly River Plains*** [32] ***(also Kimberley Plateau-Arnhem Land Barrier*** [38]***)*** *Location in this study*: A gap of up to 220 km extending inland between Port Keats and Bynoe Harbour, NT, it broadly follows the southern boundary of the Daly River drainage basin but also includes parts of the Moyle and Finniss rivers drainages. *Notes*: The second of two barriers to plant distributions identified in the Bonaparte Gulf region, the Daly River Plains is a tract of semi-arid lowland formed by the Victoria River and the headwaters of the Roper River separating the topographic relief of the Victoria River region from the Arnhem Land Plateau [39,40]. This barrier is little studied and may sometimes be confused or synonymized with the Victoria River Plains or Victoria River Drainage of Shodde & Mason [4,37].
***E*. *Arnhem Land Plateau*** *Location in this study*: An approximately 100 km disjunction running N-S that extends from the edge of the Arnhem Land escarpment east across the Arnhem Land Plateau. *Notes*: The length of this gap is hard to establish as it appears to extend beyond the southern limit of the plateau itself (at Beswick). This may be due to increasingly dry conditions extending from this point. To the north, a belt of lowlands 50 km wide between the plateau and the coast appears to allow some species to skirt this barrier. This disjunction coincides with the escarpment and western half of the Arnhem Land plateau, a massive sandstone extrusion that has been recognised as a center of endemism for both plant and animal species [41]. It has previously been indicated as a minor refugium [10], but not as a point of disjunction in the distribution of more widespread species.
***F. Carpentaria Basin (also Carpentaria Barrier*** [42]***)*** *Location in this study*: The core of this barrier is a 300 km gentle arc extending roughly SSE from the coast between the NT/QLD border and Karumba (Qld), following alluvial plains between the Selwyn Ranges and Einasleigh Uplands. *Notes*: The presence of this barrier is well recognized and appears to have existed for a long time as a filter for many species [4,36,43,44] although its width and placement has varied. While the exact cause(s) of the barrier are still unclear, aridity is usually assumed because it corresponds with an area of extensive seasonally dry alluvial plain extending inland from the Gulf of Carpentaria and separating Cape York Peninsula from the Top End region [45,46]. A plausible hypothesis for decreased precipitation is a double rain shadow with monsoonal rains blocked from the NW by Arnhemland, and SE trade winds blocked by Cape York Peninsula. It is also expected that severity of this barrier has varied across glacial cycles, with drainage of the gulf at glacial maximum allowing terrestrial species’ range expansions [43]. Despite being well recognized, descriptions of the barrier are often inconsistent or vague (particularly widths and limits of westerly extension). This may be due to a gradual environmental gradient towards its edges and the different spatial response of organisms of varying tolerances. The coastal plains continue westward for some distance in a narrowing margin along the Gulf of Carpentaria which may extend the width of this barrier for some species. It is also possible that the Barkly Tablelands to the west of the Selwyn Ranges may combine with the core barrier to form a larger gap, with some species recognizing an edaphic disjunction (sandstone ranges) and others an aridity gradient.
***G. Normanby Basin (also Normanby Barrier*** [47], ***Torresian Barrier*** [4,48,49], ***Laura Gap*** [50,51]***)*** *Location in this study*: Extending inland south-west from Princess Charlotte Bay, this gap appears to be congruent with the Normanby River basin, connecting to a corridor of lowland passing through a ~90 km gap in the Great Dividing Range north of the Einasleigh Uplands. *Notes*: A number of barriers have been invoked between Cape Melville and Cooktown and they are difficult to distinguish (e.g. Black Mountain Corridor, see below), especially as placement and width varies considerably between studies. The disjunction identified in this study best fits the concept of a broad belt corresponding to the Normanby Basin and is congruent with the Normanby Barrier of Ford [47] (also the Torresian Barrier of Schodde and Mason [48] and the Laura Gap [50,51]). It has also previously been noted that there is a “demarcation knot” across this area with an apparent interchange of tropical Asian floristic elements from the north and Sahul elements to the south [52,53].
***H*. *Black Mountain Corridor (BMC)*** *Location in this study*: Our data suggest a core gap in plant distributions of approximately 50–60 km running from between Port Douglas and Cairns inland to the Great Dividing Range *Notes*: The BMC is the widest and most consistent division between a series of isolated mesic highlands that make up the Wet Tropics [54]. It is described as an arid corridor of savannah extending from the drier inland almost to the coast, and is predicted to have been most prominent in restricting dispersal during glacial maxima [55]. While relatively well studied, few authors explicitly define limits or dimensions, instead referring to a general concept of “a gap”. This may in part be due to variation between species in width of the barrier, with various authors describing disjunctions ranging from 20 to 130 km. [55]
***I*. *Atherton Tableland*** *Location in this study*: A narrow gap approximately 30 km wide running SW-SSW from between Gordonvale and Babinda. This is broadly congruent with the high elevation of Bellenden Ker extending from the Atherton Tableland. *Notes*: Atherton Tableland and Bellenden Ker have been noted as areas of exceptionally high species diversity and endemism [56,57]. But apart from some disruption of coastal species’ ranges [58] neither have been seen as barriers to more widespread distributions (although they are partly synonymous with the eastern extremity of Ford’s Burdekin-Lynd Divide of Ford [47]). Bellenden Ker, linking with the Atherton Tableland, forms a block of habitat with exceptionally high-rainfall and elevation within the Wet Tropics. It is predicted to have been relatively stable during glacial cycles [59,60,16] which may contribute to high levels of endemicity.
***J. Townsville Lowland*** [54] ***(also Townsville Dry Corridor*** [61]***)-Paluma Range*** *Location in this study*: narrow gap of approximately 40 km in width coinciding with the Paluma Range (Spec and Halifax Uplands) between Yuruga and Toomulla and the adjacent Townsville Lowlands. *Notes*: Two locations have commonly been suggested for a biogeographical break or contact zone between Wet Tropics elements in the Ingham-Townsville area: The Herbert River Valley [47,54,62,63] narrowly separating the Kirrama and Lee uplands; and the Townsville Lowlands, a larger tract between the Herbert and Elliot Uplands[63,64]. Our results suggest a complementary barrier pair with the combined effect of the Townsville Lowlands acting as a barrier to mesic species while the wet forests of the Paluma Range Uplands acts as an immediately adjacent barrier to dry savannah adapted species. It is possible that the Herbert River Valley acts as an extension of the Townsville Lowlands, but it is unclear in our data, and the relationship between the two in previous studies is unclear.
***K. Burdekin Basin (also Burdekin Gap*** [27]***)*** *Location in this study*: A large gap of 150 km width running inland slightly south of SW, from between the Haughton River and Cape Gloucester. *Notes*: The “Burdekin Gap” has been used in many studies to describe a large break at various points between the Wet Tropics and Cape Gloucester. In a number of cases a gap with this name is poorly defined [65,66,67], is invoked to explain a much larger break between populations than the Burdekin River Basin [11,68] or is vaguely indicated as a broad swathe overlapping the Townsville Lowlands to the north and/or the Broad Sound gap to the south [69,70]. Our data suggest that, for plants, this gap is correlated with the Burdekin River Basin and is distinct from the Townsville Lowland and Broad Sound (although only approximately 50 km south of the former). Our definition is consistent with a number of previous studies suggesting a disjunction between tracts of wet forest at Paluma and Cape Gloucester and associated with reduced rainfall in the Burdekin River delta [43,71,72,73]. It has been noted that disjunctions across this region are generally subspecific in birds [27].
***L. Broad Sound*** [10] ***(also Fitzroy Gap*** [74], ***St Lawrence Gap*** [60], ***Dawson-Mackenzie Barrier*** [4]***)*** *Location in this study*: A 110 km wide disjunction intruding inland from between Cape Hillsborough and St Lawrence with no obvious geographical features as bounds. *Notes*: The first description of a “dry zone” from north of St Lawrence to Gladstone [60] is considerably wider and may have combined both the Broad Sound and Dawson-Mackenzie (below) gaps. Subsequent studies have given several names to a narrower disjunction separating the wet forest of the Clarke Range from Blackdown Tableland [4,67,75] and with “independent evolution of rainforest floristic regions north and south” [76]. This is still a wider gap than found in this study, but is broadly consistent.
***M*. *Dawson-Mackenzie*** *Location in this study*: A 45 km wide gap from just south of Gladstone to Eurimbula National Park running inland SW between Kroombit Tops and Gindoran. *Notes*: This disjunction in our data is broadly consistent with the diagrammatic representation of the Dawson-Mackenzie barrier of Schodde and Mason [4], but is not consistent with their description of it being synonymous with the Broad Sound gap of Ford [10] (which we determine to be a distinct barrier to the north). A number of other studies have indicated distributional gaps in this area, but either directly reference Broad Sound sensu Schodde and Mason [77,78] or the “St Lawrence Gap” [11] of Nix [79] which is also synonymous to Ford’s Broad Sound. Vague diagrams and no explicit definitions make it difficult to tell whether a distinct barrier is being recognized in these studies or whether there is confusion with the Broad Sound barrier further north.

#### Species distributions

Although the Australian Monsoon Tropics flora is not typically described as extending to the Pilbara region of Western Australia, we found that the Pilbara shares 714 species with the commonly accepted Australian Monsoonal Tropics. Almost 600 species have distributions spanning the entire Australian Tropics, although only two (*Aristida holathera* and *Santalum lanceolatum*) show no clear disjunctions, and no single species has disjunctions at every one of the 13 putative biogeographic barriers. 640 species were endemic to the Australian Wet Tropics.

One of the putative barriers in the Australian Monsoon Tropics (Fitzroy Basin) has greater species diversity than one of its flanking regions, but otherwise flanking regions in the Australian Monsoon Tropics are more species rich than the putative barriers (Fig 3). Little more can be made of the raw species counts because no correction was made for differences in the areas of barriers and their flanking regions. Most of the barriers are smaller in extent than their flanking areas (Fig 3).

### Hypothesis tests

At each putative barrier in the Australian Tropics, fewer species than expected by chance are disjunct (pattern "101") yet, for all but one barrier (Atherton Tableland), more species than expected by chance occur in one flanking region but not the other (Table 2). These results are consistent whether the left flank and barrier region are permuted or the right flank and barrier are permuted (see supplementary material: http://dx.doi.org/10.17632/8vgv4zzhhg.2). Our second test showed that all barriers in the Australian Monsoon Tropics are hard range-ends to many distributions (Table 2), with more species than expected by chance occurring up to, but not beyond, a barrier than expected by chance. The two putative barriers in the Australian Wet Tropics (Black Mountain Corridor and Atherton Tableland) were not hard range-ends.

**Table 2:** Results of tests of the hypotheses (1) that putative barriers are impermeable (species occur more frequently in either flank and less frequently on both sides the barrier), and (2) that species’ ranges end at a barrier more frequently than expected by chance. A plus sign (+) indicates a significantly higher frequency than expected, a minus sign (-) a significantly lower frequency than expected, and ‘ns’ indicates non-significance. As the second test was one-tailed, the only possible outcomes were ‘+’ or ‘ns’. Rows for the two barriers in the Australian Wet Tropics are shaded.

Putative Barrier	Permeability Tests			Range End Tests	
	Impermeable (100)	Permeable (101)	Impermeable (001)	Left (100)	Right (001)
A. Great Sandy Desert	+	-	+	+	+
B. Fitzroy Basin	+	-	+	+	+
C. Ord Basin	+	-	+	+	+
D. Daly River Plains	+	-	+	+	+
E. Arnhem Land Plateau	+	-	+	ns	+
F. Carpentaria Basin	+	-	+	+	+
G. Normanby Basin	+	-	+	+	+
H. Black Mountain Corridor	+	-	+	ns	ns
I. Atherton Tableland	+	-	ns	ns	ns
J. Townsville Lowlands-Paluma Range	+	-	+	+	+
K. Burdekin Basin	+	-	+	+	+
L. Broad Sound	+	-	+	+	+
M. Dawson-Mackenzie	+	-	+	+	+

## Discussion

### The Australian Monsoon tropics vs. the Australian Wet tropics

Using a comprehensive and unbiased sample of contemporary plant distributions, we have found statistical support for the conjecture that congruent disjunctions reflect real barriers to species’ range expansions across the Australian Tropics. However, the nature of barriers differs between the Australian Monsoon Tropics and the Australian Wet Tropics. In the Monsoon Tropics a significant proportion of species’ distributions are limited by 11 biogeographic barriers, with the barriers themselves being generally species-poor compared to their flanking regions, suggesting that they limit both dispersal (permeability) and colonization.

In the Australian Wet Tropics, the two disjunction zones do not limit the distributions of a significant number of species. Compared to barriers within the Australian Monsoon Tropics, they are narrower and have a higher species diversity in proportion to their flanking regions. This is consistent with the Australian Wet Tropics being comprised of a series of mesic habitat fragments weakly separated by “suture zones” or permeable barriers that are less likely to persist as continuous or contemporary distributional gaps across suites of species [16,80]. These suture zones have been described as drier corridors of mosaic wet/dry habitat extending from the interior of the continent to the coast and are partly occupied by elements of the Australian Monsoon Tropics flora, explaining their relatively high species diversity when compared to the Wet Tropics regions that flank them. This highlights a paradox when attempting to identify common distributional patterns across species assemblages: habitat that is inhospitable and a barrier to one species may be suitable habitat to other species adapted to another biome. Similarly, in the Australian Monsoon Tropics, putative barriers might harbour more arid zone flora which, in general, is more depauperate than that of more mesic biomes. Identifying weaker disjunctions remains problematic, also where species recognize different barrier widths. The methods used in this study are time consuming and analog, but more or less reliable, providing hypotheses for cross-validation using more advanced statistical tools. Recent developments in computational spatial analyses for identifying zones of biotic turnover [3,81] will be important in further quantifying and refining the extent of barriers recognized here and elsewhere.

### Taxonomy of barriers

It is hoped that our review of biogeographic barriers will stimulate clearer communication in future work on their role in the evolution of species across the Australian tropics. Of the 13 common barriers to plant distributions identified here, 11 can be directly compared to biogeographic breaks previously identified in the distributions of birds [4] and subsequently mammals [13], amphibians [82], Crustacea, and insects [12]. We have identified two novel disjunctions (Arnhem Land Plateau and Atherton Tableland) that have been previously noted as locations of high species endemism and turnover, but not as biogeographic barriers *per se* [64,83,84].

Two putative barriers identified by previous studies of the Australian Tropics (the Ord Arid Intrusion and Victoria River Drainage; [13]) were not recovered among our data. Both have been described as in close proximity to the Ord Basin and Daly River Plains barriers and failure to identify them here may be partly due to sparse sampling in that region. It was noted during scoring that a number of taxa in this area were absent across areas larger than the Ord Basin (depicted in Fig 3), consistent with a combined influence of multiple adjacent gaps—these taxa were scored as "unknown data" at these points. More specimen data are needed to resolve the biogeographic patterns across this region.

Given that most barriers identified here tend to run from the coast towards the center of the continent, it is likely that the arid interior and coastline provide bounds that prevent many species from dispersing around inhospitable habitat.

### The role of barriers in speciation and community assembly

Many previous studies have hypothesised that biogeographic barriers led to vicariance and divergence of many taxa during Pleistocene glacial phases, when climates are expected to have been significantly more arid than at present ([4,32,71,85,86], but see [87]). Expansion and renewed contact among populations after the last glacial maximum are thought to have resulted in transition zones in vertebrates between phenotypic forms that are now distributed continuously across the Australian Monsoon Tropics [44,88,89]. Thus, most recent work has concentrated on the historic role of barriers when they were supposedly at their strongest, and there is less understanding of how they continue to disrupt species distributions in the present.

Our study highlights how little is known about the nature of the barriers for Australian tropical organisms in the present, let alone the past. Questions remain about how, or whether, the nature of the barriers has changed in the glacial cycles, for example: Were there additional barriers that are not currently obvious?; or have species dispersed around or across barriers, or did these barriers emerge and divide distributions? These questions are beyond the scope of our data, and the phylogenetic relationships of the vast majority of species’ considered in this study are not well enough known to infer a direct role of barriers in speciation. A recent study by Costion *et al*. [90] found genetic divergence in multiple plant species across three barriers in the Australian Wet Tropics: 5 of 19 species across the Burdekin Basin, 37 of 110 species across the Black Mountain Corridor, and 13 of 53 species across the Normanby Basin. This indicates that some level of disruption to gene flow occurs in the vicinity of the barriers but, at this stage, it is not possible to distinguish whether these are abrupt disjunctions or the results of clinal variation [90]. Comparative analyses using molecular data within and among plant species across Australian Monsoon Tropics are clearly needed to assess the role of barriers in structuring plant populations.

Our recognition of the location and contemporary influence of barriers is a step that allows further investigation into which environmental factors may correlate with each barrier and thus could be responsible for isolating populations or communities of species. However, it is worth noting that degrees of genetic isolation between rainforest trees across fragments of the Australian Wet Tropics have been linked to fruit size and dispersal mode in otherwise ecologically similar species [91,92]. This is an important observation and highlights that climate-based ecological modeling alone cannot fully explain species’ current distributions, nor adequately predict past ranges or barriers to gene-flow [93]. Examination of traits or ecophysiological responses shared by species whose ranges end at a barrier might also provide an important insight.

Further research into the cause and effect of biogeographic barriers will need to use multiple lines of investigation, from vegetation surveys to more accurately describe barrier boundaries, to comparative phylogeographic studies for estimating past and present limits to gene flow, and ecological data to establish what environmental factors are responsible for limiting distributions and, by inference, which distributional responses may have been to historical or future climate change.

## Supporting information

S1 Document References for Fig 1. Previous literature describing or illustrating barriers or disjunctions identified in the Australian Monsoon Tropics and Australian Wet Tropics.
(DOC)
