# Supplementary material for: Congruent biogeographical disjunctions at a continent-wide scale: Quantifying and clarifying the role of biogeographic barriers in the Australian tropics
Source: PLoS One. 2017 Apr 4;12(4):e0174812. doi: 10.1371/journal.pone.0174812 (PMC5380322; doi:10.1371/journal.pone.0174812)
Supplement: S1 Document — Previous literature describing or illustrating barriers or disjunctions identified in the Australian Monsoon Tropics and Australian Wet Tropics. (DOC) [file pone.0174812.s001.doc]

SUPPLEMENTARY REFERENCES

Alacs, E. (2008) Forensics, phylogeography and population genetics: a case study using the Australasian snake-necked turtle, *Chelodina rugosa*. *PhD Thesis, University of Canberra*.

Baker, C.H., Graham, G.C., Scott, K.D., Cameron, S.L., Yeates, D.K., Merritt, D.J. (2008) Distribution and phylogenetic relationships of Australian glow-worms *Arachnocampa* (Diptera, Keroplatidae). *Molecular Phylogenetics and Evolution* **48**: 506-514.

Bee, C., Close, R. (1993) Mitochondrial DNA analysis of introgression between adjacent taxa of rock-wallabies, *Petrogale* species (Marsupialia: Macropodidae). *Genetic Research*. **61:** 21-37.

Bell, K.L., Moritz, C., Moussalli, A., Yeates, D.K. (2007) Comparative phylogeography and speciation of dung beetles from the Australian Wet Tropics rainforest. *Molecular Ecology* **16**: 4984-4998.

Bowler, J. (1976) Aridity in Australia: Age, Origins and Expression in Aeolian Landforms and Sediments. *Earth Science Review* **12**: 279-310.

Bowman, D.M.J.S., Brown, G.K., Braby, M.F., Brown, J.R., Cook, L.G., Crisp, M.D., Ford, F., Haberle, S., Hughes, J., Isagi, Y., Joseph, L., Mcbride, J., Nelson, G., Ladiges, P.Y. (2010) Biogeography of the Australian monsoon tropics. *Journal of Biogeography* **37**: 201-216.

Braby, M.F. (2008) Biogeography of butterflies in the Australian monsoon tropics. *Australian Journal of Zoology* **56**: 41-56.

Brito, P.H., Edwards, S.V. (2009) Multilocus phylogeography and phylogenetics using sequence-based markers. *Genetica*. **135:** 439-455.

Brown, G.K., Murphy, D.J., Ladiges, P.Y. (2011) Relationships of the Australo‐Malesian genus *Paraserianthes* (Mimosoideae: Leguminosae) identifies the sister group of *Acacia* sensu stricto and two biogeographical tracks. *Cladistics*.

Brown, M., Cooksley, H., Carthew, S.M., Cooper, S.J.B. (2006) Conservation units and phylogeographic structure of an arboreal marsupial, the yellow-bellied glider (*Petaurus australis*). *Australian Journal of Zoology* **54**: 305-317.

Butcher, P.A., Mcdonald, M.W., Bell, J.C. (2009) Congruence between environmental parameters, morphology and genetic structure in Australia's most widely distributed eucalypt, *Eucalyptus camaldulensis*. *Tree Genetics and Genomes* **5**: 189-210.

Cabrera, J., Jacobs, S.W.L., Kadereit, G. (2011) Biogeography of Camphorosmeae (Chenopodiaceae): tracking the Tertiary history of Australian aridification. *Telopea*. **13:** 313-326.

Catullo, R.A., Doughty, P., Keogh, J.D.R.J.S. (2011) Multi-locus phylogeny and taxonomic revision of *Uperoleia* toadlets (Anura: Myobatrachidae) from the western arid zone of Australia, with a description of a new species. *Zootaxa* **2902**: 1-43.

Chapple, D.G., Hoskin, C.J., Chapple, S.N., Thompson, M.B. (2011) Phylogeographic divergence in the widespread delicate skink (*Lampropholis delicata*) corresponds to dry habitat barriers in eastern Australia. *BMC Evolutionary Biology*. **11:** 191.

Christidis, F. (2003) Systematics, phylogeny and ecology of Australian Leptophlebiidae (Ephemeroptera). *PhD Thesis, James Cook University*.

Christidis, F., Dean, J.C. (2008) Phylogeny and distribution of the mayfly genus *Austrophlebioides* Campbell & Suter (Ephemeroptera : Leptophlebiidae). *Invertebrate Systematics* **22**: 29-36.

Colgan, D.J., O'meally, D., Sadlier, R.A. (2009) Phylogeographic patterns in reptiles on the New England Tablelands at the south-western boundary of the McPherson Macleay Overlap. *Australian Journal of Zoology* **57**: 317-328.

Cracraft, J. (1982) Geographic Differentiation, Cladistics, and Vicariance Biogeography: Reconstructing the Tempo and Mode of Evolution. *American Zoologist* **22**: 411-424.

Cracraft, J. (1986) Origin and Evolution of Continental Biotas: Speciation and Historical Congruence within the Australian Avifauna. *Evolution* **40**: 977-996.

Cracraft, J. (1991) Patterns of diversification within continental biotas: hierarchical congruence among the areas of endemism of Australian vertebrates. *Australian Systematic Botany* **4**: 211-227.

Crisp, M., Cook, L., Steane, D. (2005) Molecular dating and eucalypts: reply to Ladiges and Udovicic. *Australian Systematic Botany* **18**: 295-296.

Crome, F., Carpenter, S., Frith, H. (1980) Geographic Variation and Taxonomy of the Spinifex Pigeon, *Geophaps plumifera*. *Australian Journal of Zoology* **28**: 135-150.

Dolman, G. (2008) Evidence for Differential Assortative Female Preference in Association with Refugial Isolation of Rainbow Skinks in Australia's Tropical Rainforests. *PLoS ONE*. **3:** e3499.

Dolman, G., Moritz, C. (2006) A multilocus perspective on refugial isolation and divergence in rainforest skinks (*Carlia*). *Evolution* **60**: 573-582.

Edwards, D.L., Melville, J. (2010) Phylogeographic analysis detects congruent biogeographic patterns between a woodland agamid and Australian wet tropics taxa despite disparate evolutionary trajectories. *Journal of Biogeography* **37**: 1543–1556.

Edwards, S. (1993) Long-Distance Gene Flow in a Cooperative Breeder Detected in Genealogies of Mitochondrial DNA Sequences. *Proceedings of the Royal Society B: Biological Sciences* **252**: 177-185.

Edwards, S.V., Kot, M. (1995) Comparative methods at the species level: geographic variation in morphology and group size in grey-crowned babblers (*Pomatostomus temporalis*). *Evolution*. **49:** 1134-1146.

Eliot, I., Bird, E. (2010) *Western Australia*. In: Encyclopedia of the World's Coastal Landforms. Bird, E.(Eds.). Springer Verlag, Heidelberg.

Ford, F., Blair, D. (2005) Neat patterns with a messy history: savannah refuges in northern Australia. *Mammal Study* **30**: S45-50.

Ford, F., Johnson, C. (2007) Eroding abodes and vanished bridges: historical biogeography of the substrate specialist pebble-mound mice (*Pseudomys*). *Journal of Biogeography*. **34:** 514-523.

Ford, J. (1978) Geographical Isolation and Morphological and Habitat Differentiation Between Birds of the Kimberley and the Northern Territory. *Emu* **78**: 25-35.

Ford, J. (1979) Subspeciation, hybridization and relationships in the Little Shrike-thrush *Colluricincla megarhyncha* of Australia and New Guinea. *Emu*. **79:** 195-210.

Ford, J. (1981) Morphological and behavioural evolution in populations of the *Gerygone fusca* complex. *Emu*. **81:** 57-81.

Ford, J. (1981) Evolution, distribution and stage of speciation in the *Rhipidura fuliginosa* complex in Australia. *Emu*. **81:** 128-144.

Ford, J. (1981) Hybridization and Migration in Australian Populations of the Little and Rufus-breasted Bronze-cuckoos. *Emu*. **81:** 209-222.

Ford, J. (1982) Origin, Evolution and Speciation of Birds Specialized to Mangroves in Australia. *Emu* **82**: 12-23.

Ford, J. (1986) Avian Hybridisation and Allopatry in the Region of the Einasleigh Uplands and Burdekin-Lynd Divide, North-Eastern Australia. *Emu* **86**: 87-110.

Ford, J. (1987) Hybrid Zones in Australian Birds. *Emu* **87**: 158-178.

Ford, J. (1987) Minor Isolates and Minor Geographical Barriers in Avian Speciation in Continental Australia. *Emu* **87**: 90-102.

Fujita, M.K., McGuire, J.A., Donnellan, S.C., Moritz, C. (2010) Diversification and Persistence at the Arid-Monsoonal Interface: Australia-Wide Biogeography of the Bynoe's Gecko (*Heteronotia Binoei*; Gekkonidae). *Evolution* **64**: 2293-2314.

Gentilli, J. (1949) Foundations of Australian Bird Geography. *Emu* **49**: 85-129.

Georges, A., Doody, S., Eisemberg, C., Alacs, E. (2008) Conservation Biology of Freshwater Turtles and Tortoises. **5:** 009.001-009.017.

Greenwood, D. (1996) Eocene monsoon forests in central Australia? *Australian Systematic Botany* **9**: 95-112.

Hewitt, G.M. (2011) Quaternary phylogeography: the roots of hybrid zones. *Genetica*. **139:** 617-638.

Hilbert, D.W., Graham, A., Hopkins, M.S. (2007) Glacial and interglacial refugia within a long-term rainforest refugium: the Wet Tropics Bioregion of NE Queensland, Australia. *Palaeogeography, Palaeoclimatology, Palaeoecology*. **251:** 104-118.

Hopley, D., Smithers, S. (2010) *Queensland*. In: Encyclopedia of the World's Coastal Landforms. Bird, E.(Eds.). Springer, Verlag, Heidelberg.

Hoskin, C.J. (2007) Description, biology and conservation of a new species of Australian tree frog (Amphibia : Anura : Hylidae : *Litoria*) and an assessment of the remaining populations of *Litoria genimaculata* Horst, 1883: systematic and conservation implications of an unusual speciation event. *Biological Journal of the Linnean Society* **91**: 549-563.

Hugall, A., Moritz, C., Moussalli, A., Stanisic, J. (2002) Reconciling paleodistribution models and comparative phylogeography in the Wet Tropics rainforest land snail *Gnarosophia bellendenkerensis* (Brazier 1875). *Proceedings of the National Academy of Sciences, USA* **99**: 6112-6117.

Jacobs, S., Wilson, K. (1996) A biogeographical analysis of the freshwater plants of Australasia. *Australian Systematic Botany* **9**: 169-183.

James, C., Moritz, C. (2000) Intraspecific phylogeography in the sedge frog *Litoria fallax* (Hylidae) indicates pre-Pleistocene vicariance of an open forest species from eastern Australia. *Molecular Ecology* **9**: 349-358.

Joseph, L., Moritz, C. (1994) Mitochondrial DNA Phylogeography of Birds in Eastern Australian Rainforests: First Fragments. *Australian Journal of Botany*. **42:** 385-403.

Joseph, L., Wilke, T. (2007) Lack of phylogeographic structure in three widespread Australian birds reinforces emerging challenges in Australian historical biogeography. *Journal of Biogeography*. **34:** 612-624.

Joseph, L., Omland, K.E. (2009) Phylogeography: its development and impact in Australo-Papuan ornithology with special reference to paraphyly in Australian birds. *Emu* **109**: 1-23.

Joseph, L., Zeriga, T., Adcock, G., Langmore, N. (2011) Phylogeography and taxonomy of the Little Bronze-Cuckoo (*Chalcites minutillus*) in Australia's monsoon tropics. *Emu*. **111:** 113-119.

Joseph, L., Dolman, G., Donnellan, S., Saint, K.M., Berg, M.L., Bennett, A.T.D. (2008) Where and when does a ring start and end? Testing the ring-species hypothesis in a species complex of Australian parrots. *Proceedings of the Royal Society B: Biological Sciences***:** 2431-2440.

Kay, W., Smith, M., Pinder, A., Mcrae, J., Davis, J., Halse, S. (1999) Patterns of distribution of macroinvertebrate families in rivers of north-western Australia. *Freshwater Biology* **41**: 299-316.

Kearns, A., Joseph, L., Cook, L. (2010) The impact of Pleistocene changes of climate and landscape on Australian birds: a test using the Pied Butcherbird (*Cracticus nigrogularis*). *Emu***:** 285-295.

Kearns, A.M., Joseph, L., Omland, K.E., Cook, L.G. (2011) Testing the effect of transient Plio-Pleistocene barriers in monsoonal Australo-Papua: did mangrove habitats maintain genetic connectivity in the Black Butcherbird? *Molecular Ecology* **20**: 5042-5059.

Keast, A. (1957) Variation and speciation in the genus *Climacteris* Temminck (Aves: Sittidae). *Australian Journal of Zoology* **5**: 474-495.

Keast, A. (1958) Infraspecific Variation in the Australian Finches. *Emu*. **58:** 219-246.

Keast, A. (1961) Bird Speciation on the Australian Continent. *Bulletin of the Museum of Comparative Zoology* **123**: 307-495.

Kelemen, L., Moritz, C. (1999) Comparative phylogeography of a sibling pair of rainforest *Drosophila* species (*Drosophila serrata* and *D. birchii*). *Evolution* **53**: 1306-1311.

Kemper, C., Schmitt, L. (1992) Morphological Variation Between Populations of the Brush-Tailed Tree Rat (*Conilurus-penicillatus*) in Northern Australia and New-Guinea. *Australian Journal of Botany*. **40:** 437-452.

Kench, P. (1999) Geomorphology of Australian estuaries: Review and prospect. *Australian Journal of Ecology* **24**: 367-380.

Kidd, D.M., Ritchie, M.G. (2006) Phylogeographic information systems: putting the geography into phylogeography. *Journal of Biogeography*. **33:** 1851-1865.

Koetz, A.H., Westcott, D.A., Congdon, B.C. (2007) Geographical variation in song frequency and structure: the effects of vicariant isolation, habitat type and body size. *Animal Behaviour*. **74:** 1573-1583.

Kohlmann, B., Nix, H., Shaw, D. (1988) Environmental Predictions and Distributional Limits of Chromosomal Taxa in the Australian Grasshopper *Caledia captiva* (F.). *Oecologia*. **75:** 483-493.

Krosch, M.N. (2011) Phylogeography of *Echinocladius martini* Cranston (Diptera: Chironomidae) in closed forest streams of eastern Australia. *Australian Journal of Entomology* **50**: 258–268.

Krosch, M.N., Baker, A.M., Mckie, B.G., Mather, P.B., Cranston, P.S. (2009) Deeply divergent mitochondrial lineages reveal patterns of local endemism in chironomids of the Australian Wet Tropics. *Austral Ecology*. **34:** 317-328.

Kutt, A.S. (2004) Patterns in the composition and distribution of the vertebrate fauna, desert uplands bioregion, Queensland. *PhD Thesis, James Cook University***:** 1-85.

Ladiges, P., Ariati, S., Murphy, D. (2006) Biogeography of the *Acacia victoriae, pyrifolia* and *murrayana* species groups in arid Australia. *Journal of Arid Environments*. **66:** 462-476.

Ladiges, P., Parra-O, C., Gibbs, A., Udovicic, F., Nelson, G., Bayly, M. (2011) Historical biogeographical patterns in continental Australia: congruence among areas of endemism of two major clades of eucalypts. *Cladistics* **27**: 29-41.

Lee, J.Y., Edwards, S.V. (2008) Divergence Across Australia's Carpentarian Barrier: Stastical Phylogeography of the Red-Backed Fairy Wren (*Malurus melanocephalus*). *Evolution* **62**: 3117-3134.

Lucky, A. (2011) Molecular phylogeny and biogeography of the spider ants, genus *Leptomyrmex* Mayr (Hymenoptera: Formicidae). *Molecular Phylogenetics and Evolution*. **59:** 281-292.

MacDonald, J. (1969) Notes on the taxonomy of *Neositta*. *Emu* **69**: 169-174.

Mason, I., Forrester, R. (1996) Geographical Differentiation in the Channel-billed Cuckoo *Scythrops novaehollandiae* Latham, with Description of Two New Subspecies from Sulawesi and the Bismarck Archipelago. *Emu*. **96:** 217-233.

Matthews, E., Monteith, G.B. (2010) *Monteithum storeyi*, a new species of Adeliini (Coleoptera: Tenebrionidae) from the Wet Tropics of Queensland, Australia. *Australian Entomologist*. **36:** 201-206.

Mcdonald, M., Maslin, B. (2000) Taxonomic revision of the salwoods: *Acacia aulacocarpa* Cunn. ex Benth. and its allies (Leguminosae: Mimosoideae: section Juliflorae). *Australian Systematic Botany*. **13:** 21-78.

Mcgill, A.R. (1948) A Distributional Review of the Genus *Neositta*. *Emu*. **48:** 33-52.

Mcglashan, D., Hughes, J. (2002) Extensive genetic divergence among populations of the Australian freshwater fish, *Pseudomugil signifer* (Pseudomugilidae), at different hierarchical scales. *Marine and Freshwater Research* **53**: 897-907.

Melville, J., Ritchie, E.G., Chapple, S.N.J., Glor, R.E., Ii, J.a.S. (2011) Evolutionary origins and diversification of dragon lizards in Australia's tropical savannas. *Molecular Phylogenetics and Evolution*. **58:** 257-270.

Moran, G., Bell, J., Turnbull, J. (1989) A Cline in Genetic Diversity in River She-Oak *Casuarina cunninghamiana*. *Australian Journal of Botany*. **37:** 169-180.

Moritz, C. (1999) Conservation Units and Translocations: Strategies for Conserving Evolutionary Processes. *Hereditas*. **130:** 217-228.

Moritz, C., Hoskin, C., Graham, C.H., Hugall, A., Moussalli, A. (2005) Historical biogeography, diversity & conservation of Australia's tropical rainforest herpetofauna. *Conservation Biology Series (Cambridge)*: 243-264.

Moritz, C., Richardson, K., Ferrier, S., Monteith, G., Stanisic, J., Williams, S., Whiffin, T. (2001) Biogeographical concordance and efficiency of taxon indicators for establishing conservation priority in a tropical rainforest biota. *Proceedings of the Royal Society B: Biological Sciences* **268**: 1875-1881.

Moritz, C., Hoskin, C., Mackenzie, J., Phillips, B., Tonione, M., Silva, N., Vanderwal, J., Williams, S., Graham, C. (2009) Identification and dynamics of a cryptic suture zone in tropical rainforest. *Proceedings of the Royal Society B: Biological Sciences* **276**: 1235-1244.

Moussalli, A., Hugall, A., Moritz, C. (2005) A mitochondrial phylogeny of the rainforest skink genus *Saproscincus*, Wells and Wellington (1984). *Molecular Phylogenetics and Evolution* **34**: 190-202.

Nicholls, J., Austin, J. (2005) Phylogeography of an east Australian wet-forest bird, the satin bowerbird (*Ptilonorhynchus violaceus*), derived from mtDNA, and its relationship to morphology. *Molecular Ecology*. **14:** 1485-1496.

Nix, H. (1982) *Environment determinants of biogeography and evolution in Terra Australis*. In: Evolution of the Flora and Fauna of Arid Australia. Barker, W.R.,Greenslade, P.J.M.(Eds.). Peacock Publications, Freeville. 47-66.

Norman, J.A., Christidis, L., Joseph, L., Slikas, B., Alpers, D. (2002) Unravelling a biogeographical knot: origin of the 'leapfrog' distribution pattern of Australo-Papuan sooty owls (Strigiformes) and logrunners (Passeriformes). *Proceedings of the Royal Society B: Biological Sciences*. **269:** 2127-2133.

Oliver, P.M., Adams, M., Doughty, P. (2010) Molecular evidence for ten species and Oligo-Miocene vicariance within a nominal Australian gecko species (*Crenadactylus ocellatus*, Diplodactylidae). *BMC Evolutionary Biology* **10**: 386.

Oliver, P.M., Couper, P., Amey, A. (2010) A new species of *Pygopus* (Pygopodidae; Gekkota; Squamata) from north-eastern Queenland. *Zootaxa*. **2578:** 47-61.

Pepper, M., Doughty, P., Hutchinson, M.N., Keogh, J.S. (2011) Ancient drainages divide cryptic species in Australia's arid zone: Morphological and multi-gene evidence for four new species of Beaked Geckos (*Rhynchoedura*). *Molecular Phylogenetics and Evolution*. **61:** 810-822.

Pepper, M., Fujita, M.K., Moritz, C., Keogh, J.S. (2011) Palaeoclimate change drove diversification among isolated mountain refugia in the Australian arid zone. *Molecular Ecology*. **20:** 1529-1545.

Pepper, M., Ho, S.Y.W., Fujita, M.K., Keogh, J.S. (2011) The genetic legacy of aridification: Climate cycling fostered lizard diversification in Australian montane refugia and left low-lying deserts genetically depauperate. *Molecular Phylogenetics and Evolution*. **61:** 750-759.

Perry, R.A. (1960) *Pasture Lands of the Northern Territory Australia. CSIRO Land Research Series No.5.* . CSIRO, Melbourne.

Pope, L., Estoup, A., Moritz, C. (2000) Phylogeography and population structure of an ecotonal marsupial, *Bettongia tropica*, determined using mtDNA and microsatellites. *Molecular Ecology* **9**: 2041-2053.

Potter, S., Eldridge, M.D.B., Taggart, D.A., Cooper, S.J.B. (2012) Multiple biogeographical barriers identified across the monsoon tropics of northern Australia: phylogeographic analysis of the brachyotis group of rock-wallabies. *Molecular Ecology* **21**: 2254-2269.

Rossetto, M., Crayn, D., Ford, A., Ridgeway, P., Rymer, P. (2007) The comparative study of range-wide genetic structure across related, co-distributed rainforest trees reveals contrasting evolutionary histories. *Australian Journal of Botany* **55**: 416-424.

Rossetto, M., Crayn, D., Ford, A., Mellick, R., Sommerville, K. (2009) The influence of environment and life-history traits on the distribution of genes and individuals: a comparative study of 11 rainforest trees. *Molecular Ecology* **18**: 1422-1438.

Rowley, I. (1993) The Purple-crowned Fairy-wren *Malurus coronatus*. I. History, Distribution and Present Status. *Emu* **93**: 220-234.

Schauble, C. (2004) Variation in body size and sexual dimorphism across geographical and environmental space in the frogs *Limnodynastes tasmaniensis* and *L. peronii*. *Biological Journal of the Linnean Society* **82**: 39-56.

Schauble, C., Moritz, C. (2001) Comparative phylogeography of two open forest frogs from eastern Australia. *Biological Journal of the Linnean Society* **74**: 157-170.

Schiffer, M., Kennington, W.J., Hoffmann, A.A., Blacket, M.J. (2007) Lack of genetic structure among ecologically adapted populations of an Australian rainforest Drosophila species as indicated by microsatellite markers and mitochondrial DNA sequences. *Molecular Ecology* **16**: 1687-1700.

Schneider, C., Moritz, C. (1999) Rainforest refugia and evolution in Australia's Wet Tropics. *Proceedings of the Royal Society B: Biological Sciences*. **266:** 191-196.

Schneider, C., Cunningham, M., Moritz, C. (1998) Comparative phylogeography and the history of endemic vertebrates in the Wet Tropics rainforests of Australia. *Molecular Ecology* **7**: 487-498.

Schneider, C., Smith, T., Larison, B., Moritz, C. (1999) A test of alternative models of diversification in tropical rainforests: Ecological gradients vs. rainforest refugia. *Proceedings of the National Academy of Sciences, USA* **96**: 13869-13873.

Schodde, R. (2006) *Australia’s bird fauna today – origins and development*. In: Evolution and Biogeography of Australasian Vertebrates. Merrick, J.R., Archer, M., Hickey, G.M.,Lee, M.S.Y.(Eds.). Auscipub, Oatlands, NSW. 413–458.

Schodde, R., Mason, I. (1999) *Directory of Australian Birds. Vol. 2. Passerines*. CSIRO Publishing, Melbourne.

Shoo, L., Rose, R., Doughty, P., Austin, J.J., Melville, J. (2008) Diversification patterns of pebble-mimic dragons are consistent with historical disruption of important habitat corridors in arid Australia. *Molecular Phylogenetics and Evolution*. **48:** 528-542.

Short, L., Schodde, R., Horne, J. (1983) Five-Way Hybridization of Varied Sittellas *Daphoenositta chrysoptera* (Aves: Neosittidae) in Central Queensland. *Australian Journal of Zoology*. **31:** 499-516.

Smith, T.B., Schneider, C.J., Holder, K. (2001) Refugial isolation versus ecological gradients. *Genetica*. **112:** 383-398.

Speck, N.H., Wright, R.L., Rutherford, G.K., Fitzgerald, K., Thomas, F., Arnold, J.M., Basinski, J.J., Fitzpatrick, E.A., Lazarides, M., Perry, R.A. (1964) General Report on the Lands of the West Kimberley Area, Western Australia. *Land Research Surveys of Northern Australia, CSIRO***:** 1-228.

Toon, A., Hughes, J.M., Joseph, L. (2010) Multilocus analysis of honeyeaters (Aves: Meliphagidae) highlights spatio-temporal heterogeneity in the influence of biogeographic barriers in the Australian monsoonal zone. *Molecular Ecology* **19**: 2980-2994.

Toon, A., Mather, P., Baker, A., Durrant, K., Hughes, J. (2007) Pleistocene refugia in an arid landscape: analysis of a widely distributed Australian passerine. *Molecular Ecology* **16**: 2525-2541.

Turner, H. (1996) Sapindaceae and the biogeography of eastern Australia. *Australian Systematic Botany*. **9:** 127-132.

Van Heerwaarden, B., Kellermann, V., Schiffer, M., Blacket, M., Sgro, C.M., Hoffmann, A.A. (2009) Testing evolutionary hypotheses about species borders: patterns of genetic variation towards the southern borders of two rainforest *Drosophila* and a related habitat generalist. *Proceedings of the Royal Society B: Biological Sciences* **276**: 1517-1526.

Watson, J. (1969) Taxonomy, ecology, and zoogeography of Dragonflies (*Odonata*) from the North-west of Western Australia. *Australian Journal of Botany*. **17:** 65-112.

Webb, L.J., Tracey, J.G. (1981) *Australian rainforest: patterns and change*. In: Ecological Biogeography of Australia. Keast, A.(Eds.). Junk, The Hague, 605-694.

Whitehead, P., Bowman, D., Tideman, S. (1992) Biogeographic Patterns, Environmental Correlates and Conservation of Avifauna in the Northern Territory, Australia. *Journal of Biogeography* **19**: 151-161.

Wickneswari, R., Norwati, M. (1993) Genetic Diversity of Natural Populations of *Acacia auriculiformis*. *Australian Journal of Botany*. **41:** 65-77.

Williams, S., Hero, J. (2001) Multiple determinants of Australian tropical frog biodiversity. *Biological Conservation* **98**: 1-10.

Williams, S.E. (1997) Patterns of mammalian species richness in the Australian tropical rainforests: are extinctions during historical contractions of the rainforest the primary determinants of current regional patterns in biodiversity? *Wildlife Research*. **24:** 513-530.

Williams, S.E., Pearson, R.G. (1997) Historical rainforest contractions, localized extinctions and patterns of vertebrate endemism in the rainforests of Australia's wet tropics. *Proceedings of the Royal Society B: Biological Sciences*. **264:** 709-716.

Williams, S.E., Pearson, R.G., Walsh, P.J. (1996) Distributions and biodiversity of the terrestrial vertebrates of Australia's Wet Tropics: a review of current knowledge. *Pacific Conservation Biology*. **2:** 327-362.

Winter, J. (1997) Responses of non-volant mammals to late Quaternary climatic changes in the wet tropics region of north-eastern Australia. *Wildlife Research*. **24:** 493-511.

Winter, J.W., Bell, F.C., Pahl, L.I., Atherton, R.G. (1984) *The specifc habitats of selected northeastern Australian rainforest mammals. Report to the World Wildlife Fund, Sydney, Australia.*,

Woinarski, J.C.Z., Hempel, C., Cowie, I., Brennan, K., Kerrigan, R., Leach, G., Russell-Smith, J. (2006) Distributional pattern of plant species endemic to the Northern Territory, Australia. *Australian Journal of Botany* **54**: 627–640.

Wong, B.B.M., Keogh, J.S., Mcglashan, D.J. (2004) Current and historical patterns of drainage connectivity in eastern Australia inferred from population genetic structuring in a widespread freshwater fish *Pseudomugil signifer* (Pseudomugilidae). *Molecular Ecology* **13**: 391-401.

Zeisset, I., Beebee, T.J.C. (2008) Amphibian phylogeography: a model for understanding historical aspects of species distributions. *Heredity*. **101:** 109-119.
